# Envisioning sustainable and equitable World Health Assemblies

**DOI:** 10.1136/bmjgh-2022-009231

**Published:** 2022-05-25

**Authors:** Parnian Khorsand, Maisoon Chowdhury, Arthur Wyns, Lotta Velin, Marie-Claire Wangari, Gabriela Cipriano, Omnia El Omrani, Poorvaprabha Patil, Kim van Daalen

**Affiliations:** 1 Women in Global Health, Washington, DC, USA; 2 Climate and Health Alliance, Wadawurrung Country, Victoria, Australia; 3 Centre for Teaching and Research in Disaster Medicine and Traumatology, Linkoping, Sweden; 4 Wajibu Wetu Center, Nairobi, Kenya; 5 Cayetano Heredia University, Lima, Peru; 6 Plastic, Reconstructive and Aesthetic Surgery Department, Ain Shams University Teaching Hospital, Cairo, Egypt; 7 Kasturba Medical College Manipal, Manipal, Karnataka, India; 8 Cardiovascular Epidemiology Unit, Department of Public Health and Primary Care, University of Cambridge, Cambridge, Cambridgeshire, UK

**Keywords:** Environmental health, Health policy, Public Health

**Summary box**

- Over the past two years, the global health community worked, primarily virtually, to coordinate historic efforts in response to the COVID-19 pandemic, challenging the notion that the ‘pre-pandemic’ financially and environmentally costly business and travel would be essential for global health decision-making to be successful.
- Participation in large global health meetings, such as the World Health Assemblies (WHAs), has historically been inequitable, with limited representation of attendees from specific geographical locations, and those from certain socioeconomic, gender and ethnic backgrounds.
- Growing literature has explored the enormous amount of greenhouse gas emissions (and accompanying air pollution) from conferences. Despite the global push for sustainability in global health conferences, roundtrip travel alone to WHA72 (2019) has been estimated to result in 2127 tonnes CO_2_ emissions (0.84 tonnes CO_2_ per capita). This is almost equivalent to the 2020 per capita CO_2_ emissions of Nicaragua(0.70 tonnes CO_2_), Papua New Guinea (0.83 tonnes CO_2_) and Djibouti(0.85 tonnes CO_2_).
- Virtual or hybrid format conferences do not preclude inequities, may still have a significant environmental impact, and, importantly, have the potential to further accentuate inequities if not mindfully planned. Reflection regarding the mechanisms under which WHA is currently organised and the size of Member State delegations and their contributions to GHG emissions is required.

Prior to the pandemic, in-person international conferences and meetings held a fundamental role in intergovernmental and multistakeholder decision-making, advocacy and networking within the global health landscape, but COVID-19 forced borders to close, and for diplomacy and decision-making to thus take place virtually.^1^ This switch to virtual environments has enabled broader access to diverse voices not typically represented at the conference table, reduced cost barriers and visa requirements, and exemplified the efficiency and effectiveness of virtual meetings.

One of the most central global health meetings within the field is the World Health Assembly (WHA); the decision-making body of the WHO, at which a majority of the global health policies are agreed on and set. The annual WHA enables Member States, non-governmental organisations and various other non-state actors to convene for the purpose of taking formal decisions on the core values, goals and programmes of WHO’s work going forward. In the year 2020, WHA took place largely virtually, and in 2021, a hybrid model was implemented, raising an important question: if it is possible for the world to coordinate global health efforts virtually, is it necessary to return to the ‘pre-pandemic status quo’ volume of financially and environmentally costly business and travel for global health decision-making to be successful?

Even though a significant portion of the Global South continues to face inequitable access to and the ability to safely deliver COVID-19 vaccines, in-person meetings are reconvening. With the rampant disparity in vaccination rates across the world (with 79% of people in the USA and Canada being at least partially vaccinated, but only 21% in the African continent being partially vaccinated including countries with 0.1% vaccination of their population—as of 6 May 2022),^2^ resuming pre-pandemic travel patterns may further exclude already under-represented populations and further extend such inequity. Compounding to the global vaccine inequities, individuals who are not fully vaccinated or those who have not received vaccines authorised in Switzerland (ie, Moderna, Pfizer/BionTech or Janssen) may be unable to travel into the country.^3^ Therefore, it is imperative to re-evaluate the significance, necessity and accessibility of travel for global health governance; especially when decisions are made that will largely impact the countries who are unable to be present at the table.

## Inequity in the participation of in-person conferences

Travel to and from conferences is typically both expensive and carbon-intensive, often dominated by the Global North, perpetuating existing power imbalances within global health, and possibly leaving a higher burden on the shoulders of attendees from specific geographical locations and socioeconomic, gender and ethnic backgrounds.^4 5^ At the same time, the carbon-intensive nature of this travel raises questions around planetary health impacts.^4^ Participation in global health convenings, such as WHAs over the past 74 years, has historically been inequitable, with limited representation of attendees from specific geographical locations—particularly lower-middle income countries (LMICs)—and those from certain socioeconomic, gender and ethnic backgrounds.^1^ Illustratively, a recent review of 112 global health conferences found that LMIC attendees are often under-represented in global health conferences due to systematic barriers that include visa restrictions (disproportionately stringent requirements and complex procedures), financial barriers (high overall costs of travel, stay and visas), political barriers (eg, corruption at local embassies, regional conflicts or epidemics, the fear of host countries that attendees do not plan to leave), or cases of discrimination and racism (on the basis of nationality and ‘weaker’ passport status).^1^ These findings can similarly be extended to participation at the WHA, annually held in Geneva, Switzerland, where attendees from approximately 150 countries require a visa,^6^ and significant financial means for travel and subsistence for up to 2 weeks, in a city that ranks seventh in the top 10 of the Worldwide Cost of Living Index.^7^ In particular, attendees from LMICs disproportionately have to undergo time-intensive and costly processes when applying for a visa, without having any guarantee that the visa will be granted or that there is an opportunity to re-apply.

## On the planetary health impacts

Growing literature^8^ has explored the enormous amount of greenhouse gas (GHG) emissions (and the accompanying air pollution) from academic and political conferences. Emissions are generated by both air and land travel, hospitality services, single-use conference items (eg, badges, water bottles), the provision of unsustainable dietary choices in the conference menus,^9^ manufacturing of conference items (eg, tote bags, USB sticks), the increased use of electricity and use of audio-visual equipment.^10^ Nevertheless, a large portion of these emissions can be traced back to travel, including modes, frequency and distance. For example, when modelling the travel reduction potentials of three global conferences on the subject of ecology, travel emissions averaged 722–955 t CO_2_e per conference and 1.3–1.8 t CO_2_e per attendee.^11^ This striking amount is almost half of the per capita CO_2_ emission that Switzerland produces annually,^12^ and far exceeds the annual per capita amount of many LMICs. Our analysis estimated the CO_2_ emissions produced solely by WHO Member State delegations assumed air transport to the WHA by inferring their flight path and corresponding emissions (methods in the online supplemental materials), based on publicly available data on WHA delegations collected by Women in Global Health. For the 2019 WHA 72, we found the total emission to be 2127 tonnes CO_2_, with an average of 0.84 tonnes CO_2_ per delegate (see online supplemental materials and online supplemental table 1 for the methodology and details). This is almost equivalent to the 2020 per capita CO_2_ emissions of Nicaragua (0.70 tonnes CO_2_), Papua New Guinea (0.83 tonnes CO_2_) and Djibouti (0.85 tonnes CO_2_).^13^

10.1136/bmjgh-2022-009231.supp1 Supplementary data

These emissions are significant, as human-induced climate change has caused and is continuing to cause widespread harm to people and the planet.^14^ This includes the increasing impacts on human physical and mental health, and well-being globally.^15^ Extreme weather and climate events have displaced hundreds of thousands of people, and resulted in the death of millions of humans and animals per year,^15^ many of the topics which are discussed annually at global meetings such as the WHA.^16^ Likewise, the changing climate alters environmental susceptibility for infectious disease transmission (eg, malaria, chikungunya, West Nile virus, vibrio). Moreover, threats to food and water security undermine the social and physical determinants of good health.^17 18^ Importantly, the effects of climate change on health—both incremental and disastrous—are often unequal.^19 20^ The populations being disproportionately impacted have historically contributed the least to the problem, and largely comprise racial and ethnic minorities including Indigenous people, women and gender minorities, and the Global South.^20^ This dynamic is mirrored by ‘global health meeting inequity’, where those that often suffer from the largest proportion of the global burden of disease are under-represented at global health fora and decision-making tables, such as the WHA. This inequity hence perpetuates existing power imbalances within global health and reveals deeper questions of injustice.

## Virtual and hybrid models: equity friend or foe?

In the wake of the COVID-19 pandemic, many advocates and researchers^21^ have called for a fully or partly digital format of global health political and academic fora to reduce emissions and increase equitable participation. Yet, a completely digital format of the WHA is likely to disproportionately limit participation, engagement and policy influence of non-state stakeholders, including youth and other members of civil society, and those traditionally under-represented.^22^ Arguably, the WHA serves a broader function than exclusively decision-making through the formal proceedings, including a significant political and social function. A wide range of formal, semiformal and informal activities bring government, community-based organisations and non-governmental organisations (NGOs) together through technical briefings, side events, receptions and ad hoc meetings.^22^ These programmes are often key moments for civil society (CSO) and other non-state organisations to engage in the governing body meetings, and share their technical expertise and lived experiences. They further provide opportunities for networking and personal development, which can be significant in career progression. However, online formats limit the ability to facilitate these interactions, reducing the space for CSO engagement at the WHA—a space that is already seemingly shrinking, as expressed by representatives of CSO.^23^

Furthermore, while online formats remove the need to travel, they do not preclude inequities in access, with barriers such as limited strong broadband networks required for participation, English as the predominant language, time zone differences, limited digital literacy and balancing caregiving responsibilities. These factors are likely to disproportionately hinder participation of LMIC participants. Nearly half of the world’s population is still lacking access to the required technology and infrastructure for online participation, the so-called digital divide.^24^ Inequities in meaningful participation may also be exacerbated due to the different norms of interactions in an online format compared with in-person communication, making it more challenging for under-represented, younger and junior-level participants to engage and establish themselves within a network of colleagues.^21^ Likewise, hybrid conferences may further exacerbate inequities in participation and power dynamics. This could be the case if it becomes the norm that certain attendees only participate online, while others are able to participate in person (eg, high-income countries(HICs) vs LMICs, or Member States vs Civil Society), significantly reducing the interaction between certain actors and limiting the influence of civil society groups on WHA processes. Moreover, even though online fora create less GHG emissions compared with in-person events, they still contribute a significant environmental impact resulting from energy use (eg, network data transfer, server, electricity) and computer life cycle emissions.^25^ A balance must be struck so that the fora can be held equitably but with a smaller ecological footprint.

## The role of the WHA and Member States

The WHO, its Member States and CSOs can play an instrumental role in ensuring that the WHA and other global health meetings become more sustainable and equitable. In terms of sustainability, the WHO has been reporting its institutional emissions to the UN since 2009, but environmental monitoring has been limited to selected activities at headquarters, and does not include regional or country offices, nor WHO meetings such as WHA or Executive Board meetings.^26^ Meanwhile, the WHO could put transparent and detailed systems in place to track various data on the WHA. This can include, for example, tracking the number of registered delegates physically present at the event, travel and visa costs per delegate, and the time taken for visa processes. The WHO should reassess the necessity to host the annual meetings strictly in Geneva, Switzerland, an expensive and difficult to access place for the majority of Member States, who are unable to access direct, affordable or any flights at all (see online supplemental materials and online supplemental table 1). The WHO is one of only a few outstanding UN agencies that have not yet developed an institutional climate mitigation strategy.^27^ Furthermore, the WHO has a set of internal Environmental Management Procedures (EMPs), but these have had no significant effect on the organisation’s overall GHG emissions thus far.^27^ The EMPs also lack transparency and are limited to the Geneva Headquarters. In 2021, a small group of WHO staff and the WHO staff association voluntarily set up an initiative called ‘Greening WHO,’ but there has been limited support from the organisation’s central management. Lastly, when looking at equitable participation, the WHO has recently initiated a Diversity Equity and Inclusion (DEI) Initiative, aimed at developing the organisation’s first-ever DEI strategy, to be developed and supported by a Global Advisory Group and Regional Catalyst Groups, alongside DEI workshops and staff support. Yet, thus far no information has been published on the scope of the DEI strategy, and it is unclear at this stage whether it will provide guidance on equitable participation at the WHA.

## Conclusion, steps forward and recommendations

There is an imperative to reassess the ways in which the WHA functions to ensure both equitable and meaningful participation in global health decision-making, and reduce the environmental impact of this annual forum. Lessons learnt during this reassessment process can be further applied across UN institutions, and political or academic convenings more broadly. In its current state, the organisation of the WHA has a significant carbon footprint. To contribute to GHG emission reduction and commit to combat climate change in line with the Paris Agreement, it is therefore imminent to reduce the WHA emissions and to ‘walk the talk’ in the transition towards more sustainable and healthy societies. At the same time, it needs to be recognised that global health fora have traditionally been, and continue to be inequitable. The recent hiatus on in-person meetings allows us to reconsider what the global health community wants the WHA to look like and to represent.

Our aim with this piece is not to provide a comprehensive resource with recommendations, rather we hope it will start a long overdue conversation and call for action to reflect on the way the WHA is currently organised and explore whether the different formats—online and/or hybrid—when adopted, can contribute to reducing carbon emissions while ensuring more equitable participation. Furthermore, it will provide the global health community an opportunity to reflect on how WHA processes must align with the WHO’s *‘*triple billion’ goal.^28^ We provide a few potential guiding recommendations for the WHO that could lead to more sustainable and equitable WHAs, which by extension may also apply to other meetings organised by the WHO, and the WHO more broadly (box 1). We welcome suggestions and recommendations from policymakers and civil society around the globe, especially from those often left out at the decision-making table.

Box 1 **Potential recommendations for the WHO towards the organisation of more equitable and sustainable WHAs and global health meetings.**

1. Adopt and implement an institutional roadmap towards carbon neutrality by 2030, in line with proposals made by the WHO’s own staff as part of the LEAD Challenge.
2. Initiate an environmental management group to implement its carbon neutrality roadmap, and which coordinates activities across all levels of the organisation: Headquarters, Regional Offices and Country offices.
3. Develop and implement a policy on sustainable and equitable global health conferences, that facilitates meaningful participation of those most impacted and least represented groups and Members at the WHA.
4. Develop a strategy on diversity, equity and inclusion to include DEI measures for WHO’s conferences, including the WHA.
5. Create a transparent system of reporting the climate impact and inclusivity of its events, specifically the WHA and the WHO Executive Board (eg, including reporting NGO/CSO delegates’ location (country);
6. Host the WHA at locations beyond Geneva, potentially utilising its regional office locations, to enable easier access for countries outside of North America and Europe.
7. Divest from fossil fuels for all its financial assets.
8. While taking into account that the primary focus should be the reduction of GHG emissions: adopt and implement meaningful and effective solutions that aim to transparently reduce ‘inevitable’ carbon emissions, including by offsetting travel, energy and conference-specific emissions from WHA through accredited schemes that are able to deliver real, measurable and additional emission reductions. (Note: It should be acknowledged that carbon offsetting remains controversial as this has been used in the past to justify unsustainable practices.)

## Data Availability

All data relevant to the study are included in the article and in the supplement materials.
