# Supplementary material for: Envisioning sustainable and equitable World Health Assemblies
Source: BMJ Glob Health. 2022 May 25;7(5):e009231. doi: 10.1136/bmjgh-2022-009231 (PMC9134203; doi:10.1136/bmjgh-2022-009231)
Supplement: Supplementary data [file bmjgh-2022-009231supp001.pdf]

Supplement Materials

Ethical Considerations

All data used for this study was not restricted nor sensitive, nor did it require permission to access or collate. Data was publicly available and accessible, eliminating the need for additional ethical approval.

Data sources

To obtain the total number of delegates for each Member State delegation in 2019, the official lists of delegates and other participants were obtained from the WHO's Institutional Repository for Information Sharing (IRIS) (https://apps.who.int/iris/) for the 72nd World Health Assembly May 20th to May 28th, 2019 (https://apps.who.int/gb/ebwha/pdf_files/WHA72/A72_Div1Rev1-en.pdf). WHA72 was utilised, as both WHA73 and WHA74 took place (largely) virtually due to the COVID-19 pandemic, which impacted delegation size and travel. Notably, across countries, there is variability in delegation size (e.g. Belgium 14 delegates compared to China 106 delegates). According to Article 11 of the WHO Constitution, all Member States can have a maximum of three delegates. However, they can be accompanied by advisors and alternatives - resulting in a wide range of delegation sizes.

Delegates representing civil society or other non-governmental organisations (CSO) were not included, as there is no information in the official lists of delegates pertaining to their location in the world, complicating our ability to determine their likely travel paths and greenhouse gas (GHG) emissions accordingly. However, some country delegations may still contain CSO or NGO experts.

Calculating the overall GHG emissions per delegation

Khorsand P, *et al. BMJ Global Health* 2022; 7:e009231. doi: 10.1136/bmjgh-2022-009231

A flight path was determined for a delegate traveling from their respective Member State capital to Geneva, Switzerland using Skyscanner (https://www.skyscanner.com) and Google Flights (https://www.google.com/flights/). In case there was no flight available from the capital to Geneva, we used the suggested best path by Skyscanner and Google Flights as flight path. Under the assumption delegates fly Economy, but opting for the shortest distance possible, this flight path (city of origin, connecting cities, and city of destination) was then inserted into the ICAO Carbon Emissions Calculator to calculate an estimate of the carbon emissions of each passenger traveling roundtrip to and from Geneva, Switzerland.(https://www.icao.int/environmental-protection/Carbonoffset/Pages/default.aspx). This calculator utilizes the best publicly available data to enable the consideration of various factors including e.g. aircraft type, cargo carried and route specific data. The calculated CO2 emissions take into consideration the load factor and are only based on passenger operations (e.g. fuel burn related to belly freight is not included). The obtained carbon emission estimation per Member State delegate was then multiplied by the number of delegates attending on behalf of that Member State indicated in the World Health Assembly list of delegates.

Neither Skyscanner nor Google flights showed any results for flight searches from the following cities to Geneva, Switzerland. Our estimates for these countries were calculated based on their closest neighbouring country.

- Thimphu, Bhutan
- Mazini, Eswatini
- South Tarawa, Kiribati
- Yaren, Nauru
- Dili, Timor Leste

- San’a, Yemen

Caveats

- The ICAO calculator is limited to calculating CO_2 amounts released.
- The ICAO calculator uses only a few generic types of aircrafts, instead of the one specifically operating the relevant flight

References:

1. Irwin R. Reforming the World Health Assembly. BMJ Glob Heal. 2020;5(5):e002570. https://doi.org/10.1136/BMJGH-2020-002570

Supplementary Table 1. Estimated CO_2 emissions from travel of country delegations to the 72nd World Health Assembly (2019)

Country	# Delegates	Flight route	CO2 KG/ Roundtrip for total Delegates	CO2 KG/ Passenger Roundtrip
Afghanistan	11	KBL Kabul > IST Istanbul > GVA Geneva	7229.2	657.2
Albania	7	TIA Tirana > GVA Geneva	1451.8	207.4
Algeria	10	ALG Algiers > GVA Geneva	3400	340
Andorra	1	GIR Girona > AMS Amsterdam > GVA Geneva	426.8	426.8
Angola	19	LAD Luanda > LIS Lisbon > GVA Geneva	18787.2	988.8
Antigua and Barbuda	2	ANU Saint John's > LGW London > GVA Geneva	2105.2	1052.6
Argentina	10	EZE Buenos Aires > MAD Madrid > GVA Geneva	10901	1090.1
Armenia	9	EVN Yerevan > VIE Vienna > GVA Geneva	5576.4	619.6
Australia	13	CBR Canberra > MEL Melbourne > AUH Abu Dhabi > GVA Geneva	22521.2	1732.4
Austria	11	VIE Vienna > GVA Geneva	2420	220
Azerbaijan	6	GYD Baku > IST Istanbul > GVA Geneva	3808.8	634.8
Bahamas	8	NAS Nassau > YYZ Toronto > YUL Montreal > GVA Geneva	9013.6	1126.7
Bahrain	9	BAH Al Muhharaq > IST Istanbul > GVA Geneva	6141.6	682.4
Bangladesh	23	DAC Dhaka > DXB Dubai > GVA Geneva	24016.6	1044.2
Barbados	4	BGI Bridgetown > YYZ Toronto > YUL Montreal > GVA Geneva	5272.8	1318.2
Belarus	11	MSQ Minsk > FRA Frankfurt > GVA Geneva	5658.4	514.4
Belgium	14	BRU Brussels > GVA Geneva	2133.6	152.4
Belize	2	BZE Ladyville > MIA Miami > LHR London > GVA Geneva	2428	1214
Benin	11	COO Cotonou > ABJ Abidjan > IST Istanbul > GVA Geneva	12597.2	1145.2
Bhutan	11	DEL New Delhi > MUC Munich > GVA Geneva	6415.2	583.2
Bolivia	12	LPB La Paz > VVI Santa Cruz > GRU Sao Paulo> CDG Paris > GVA Geneva	17026.8	1418.9
Bosnia and Herzegovina	3	SJJ Sarajevo > FRA Frankfurt > GVA Geneva	1216.2	405.4
Botswana	20	GBE Gaborone > JNB Johannesburg > DXB Dubai > GVA Geneva	32112	1605.6

Brazil	38	BSB Brasilia > GIG Rio De Janeiro > MAD Madrid > GVA Geneva	45645.6	1201.2
Brunei Darussalam	8	BWN Bandar Seri Begawan > KUL Kuala Lumpur > AUH Abu Dhabi > GVA Geneva	10480	1310
Bulgaria	5	SOF Sofia > FRA Frankfurt > GVA Geneva	2346	469.2
Burkina Faso	11	OUA Ouagadougou > ACC Accra > CDG Paris > GVA Geneva	10312.5	937.5
Burundi	18	BJM Bujumbura > KGL Kigali > EBB Entebbe > BRU Brussels > GVA Geneva	12513.6	695.2
Cambodia	7	PNH Phnom Penh > KUL Kuala Lumpur > DXB Dubai > GVA Geneva	10403.4	1486.2
Cameroon	8	NSI Yaoundé > DLA Douala > ADD Addis Ababa > GVA Geneva	8814.4	1101.8
Canada	27	YOW Ottawa > YYZ Toronto > LGW London > GVA Geneva	22790.7	844.1
Cape Verde	5	RAI Praia > LIS Lisbon > GVA Geneva	3592.5	718.5
Central African Republic	8	BGF Bangui > NSI Yaoundé > CDG Paris > GVA Geneva	8099.2	1012.4
Chad	8	NDJ N'Djamena > ABV Abuja > CDG Paris > GVA Geneva	7110.4	888.8
Chile	10	SCL Santiago > MAD Madrid > GVA Geneva	11368	1136.8
China	106	PEK Beijing > HEL Helsinki > BRU Brussels > GVA Geneva	100636.4	949.4
Colombia	12	BOG Bogota > FRA Frankfurt > GVA Geneva	13291.2	1107.6
Comoros	7	HAH Moroni > ADD Addis Ababa > GVA Geneva	6041	863
Congo, Democratic Republic of	15	FIH Kinshasa > IST Istanbul > GVA Geneva	14490	966
Cook Islands	3	RAR Rarotonga > AKL Auckland > LAX Los Angeles > ZRH Zúrich > GVA Geneva	7253.7	2417.9
Costa Rica	8	SJO San José > MAD Madrid > GVA Geneva	9257.6	1157.2
Cote d'Ivoire	14	ABJ Abidjan > CDG Paris > GVA Geneva	10385.2	741.8
Croatia	7	ZAG Zagreb > VIE Vienna > GVA Geneva	2234.4	319.2
Cuba	11	HAV Havana > MAD Madrid > GVA Geneva	11234.3	1021.3
Cyprus	8	LCA Larnaca > VIE Vienna > GVA Geneva	4584	573
Czech Republic	12	PRG Prague > GVA Geneva	2181.6	181.8
Denmark	21	CPH Copenhagen > GVA Geneva	5598.6	266.6
Djibouti	8	JIB Djibouti > ADD Addis Ababa > GVA Geneva	5580	697.5
Dominica	8	DOM Dominica > FDF Fort De France > ORY Paris > GVA Geneva	7579.2	947.4
Ecuador	8	UIO Quito > PTY Panama City > FRA Frankfurt > GVA Geneva	10840	1355
Egypt	15	CAI Cairo > GVA Geneva	5994	399.6
El Salvador	4	SAL San Salvador > MAD Madrid > GVA Geneva	2857.2	714.3

Equatorial Guinea	6	SSG Malabo > LOS Lagos > FRA Frankfurt > GVA Geneva	5485.2	914.2
Eritrea	6	ASM Asmara > ADD Addis Ababa > GVA Geneva	4201.2	700.2
Estonia	7	TLL Tallinn > FRA Frankfurt > GVA Geneva	3238.2	462.6
Eswatini (Swaziland)	7	CPT Cape Town > ADD Addis Ababa > GVA Geneva	9053.8	1293.4
Ethiopia	18	GDQ Gondar > ADD Addis Ababa > GVA Geneva	11156.4	619.8
Fiji	5	SUV Suva > NAN Nan > SYD Sydney > DXB Dubai > GVA Geneva	12708	2541.6
Finland	12	HEL Helsinki > MUC Munich > GVA Geneva	5553.6	462.8
France	35	ORY Paris > GVA Geneva	4081	116.6
Gabon	9	LBV Libreville > CDG Paris > GVA Geneva	7612.2	845.8
Gambia	21	BJL Banjul > DSS Dakar > BRU Brussels > GVA Geneva	15981	761
Georgia	9	TBS Tbilisi > KBP Kiev > GVA Geneva	6157.8	684.2
Germany, Federal Republic of	22	BER Berlin > GVA Geneva	4501.2	204.6
Ghana	37	ACC Accra > LHR London > GVA Geneva	32256.6	871.8
Greece	8	ATH Athens > GVA Geneva	2452.8	306.6
Grenada	8	GND St. George > JFK New York > BCN Barcelona > GVA Geneva	11020.8	1377.6
Guatemala	8	GUA Guatemala City > SAL San Salvador > MAD Madrid > GVA Geneva	6476.8	809.6
Guinea	9	CKY Conakry > CDG Paris > GVA Geneva	6513.3	723.7
Guinea Bissau	3	OXB Bissau > LIS Lisbon > GVA Geneva	2130.6	710.2
Guyana	6	GEO Georgetown > JFK New York > MAD Madrid > GVA Geneva	7944.6	1324.1
Haiti	11	PAP Port Au Prince > BOS Boston > LHR London > GVA Geneva	13303.4	1209.4
Holy See	7	FCO Rome > GVA Geneva	1223.6	174.8
Honduras	6	TGU Tegucigalpa > SAL San Salvador > MAD Madrid > GVA Geneva	4882.2	813.7
Hungary	8	BUD Budapest > GVA Geneva	1729.6	216.2
Iceland	4	KEF Reykjavik > ORY Paris > GVA Geneva	1882.4	470.6
India	34	DEL New Delhi > MUC Munich > GVA Geneva	19828.8	583.2
Indonesia	64	CGK Jakarta > DXB Dubai > GVA Geneva	89164.8	1393.2
Iran, Islamic Republic of	17	IKA Tehran > VIE Vienna > GVA Geneva	11362.8	668.4
Iraq	6	SDA Baghdad > IST Istanbul > GVA Geneva	3379.2	563.2

Khorsand P, *et al. BMJ Global Health* 2022; 7:e009231. doi: 10.1136/bmjgh-2022-009231

Ireland	9	DUB Dublin > GVA Geneva	2149.2	238.8
Israel	8	TLV Tel Aviv > GVA Geneva	3259.2	407.4
Italy	22	FCO Rome > GVA Geneva	3845.6	174.8
Jamaica	10	KIN Kingston > YYZ Toronto > LIS Lisbon > GVA Geneva	13654	1365.4
Japan	33	HND Tokyo > IST Istanbul > GVA Geneva	35590.5	1078.5
Jordan	4	AMM Amman > VIE Vienna > GVA Geneva	2331.2	582.8
Kazakhstan	17	NQZ Nursultan > SVO Moscow > GVA Geneva	10693	629
Kenya	52	NBO Nairobi > FRA Frankfurt > GVA Geneva	47840	920
Kiribati	7	SUV Suva > NAN Nan > SYD Sydney > DXB Dubai > GVA Geneva	17791.2	2541.6
Kuwait	11	KWI Kuwait > FRA Frankfurt > GVA Geneva	7317.2	665.2
Kyrgyzstan	4	FRU Bishkek > IST Istanbul > GVA Geneva	2683.2	670.8
Lao People's Democratic Republic	13	VTE Vientiane > BKK Bangkok < VIE Vienna < GVA Geneva	14042.6	1080.2
Latvia	6	RIX Riga > FRA Frankfurt > GVA Geneva	2526	421
Lebanon	10	BEY Beirut > FRA Frankfurt > GVA Geneva	5622	562.2
Lesotho	9	MSU Maseru > JNB Johannesburg > FRA Frankfurt > GVA Geneva	14065.2	1562.8
Liberia	5	ROB Monrovia > BKO Bamako > CDG Paris > GVA Geneva	3477	695.4
Libya	12	MJI Tripoli > TUN Tunis > GVA Geneva	4670.4	389.2
Lithuania	5	VNO Vilnius > FRA Frankfurt > GVA Geneva	2092	418.4
Luxembourg	9	LUX Luxembourg > GVA Geneva	1004.4	111.6
Madagascar	11	TNR Antananarivo > MRU Sir Seewoosagur Ramgoolam > DXB Dubai > GVA Geneva	16479.1	1498.1
Malawi	9	LLW Lilongwe > ADD Addis Ababa > GVA Geneva	7769.7	863.3
Malaysia	17	KUL Kuala Lumpur > AUH Abu Dhabi > GVA Geneva	18098.2	1064.6
Maldives	7	MLE Huhule Island > IST Istanbul > GVA Geneva	5943	849
Mali	22	BKO Bamako > CDG Paris > GVA Geneva	12394.8	563.4
Malta	6	MLA Luqa > MUC Múnich > GVA Geneva	2640	440
Marshall Islands	4	MAJ Marshall Islands > KWA Kwajalein > PNI Pohnpei > TKK Chuuk > GUM Guam > NRT Narita > ZRH Zurich > GVA Geneva	7531.6	1882.9
Mauritania	13	NKC Nouakchott > DSS Dakar > IST Istanbul > GVA Geneva	14021.8	1078.6
Mauritius	6	MRU Mauritius > DXB Dubai > GVA Geneva	7788	1298

Mexico	19	MEX Mexico City > AMS Amsterdam > GVA Geneva	20615	1085
Micronesia, Federated States of	1	PNI Pohnpei > TKK Truk > GUM Guam > ICN Seoul > AMS Amsterdam > GVA Geneva	1670.6	1670.6
Moldova	4	KIV Chisinau > VIE Vienna > GVA Geneva	1821.6	455.4
Monaco	12	NCE Nice > GVA Geneva	1219.2	101.6
Mongolia	11	UBN Ulaanbaatar > IST Istanbul > GVA Geneva	9352.2	850.2
Montenegro	6	TGD Podgorica > VIE Vienna > GVA Geneva	2394	399
Morocco	13	RBA Rabat > CDG Paris > GVA Geneva	5969.6	459.2
Mozambique	10	MPM Maputo > ADD Addis Ababa > GVA Geneva	10759	1075.9
Myanmar	12	RGN Yangon > BKK Bangkok > VIE Vienna > GVA Geneva	13663.2	1138.6
Namibia	13	WDH Windhoek > FRA Frankfurt > GVA Geneva	13824.2	1063.4
Nauru	3	HND Tokyo > IST Istanbul > GVA Geneva	3235.5	1078.5
Nepal	11	KTM Kathmandu > DOH Hamad > LHR London > GVA Geneva	13586.1	1235.1
Netherlands	23	AMS Amsterdam > GVA Geneva	4457.4	193.8
New Zealand	7	WLG Wellington > AKL Auckland > SIN Singapore > FRA Frankfurt > GVA Geneva	14102.9	2014.7
Nicaragua	4	MGA Managua > MIA Miami > FRA Frankfurt > GVA Geneva	5741.2	1435.3
Niger	6	NIM Niamey > ADD Addis Ababa > GVA Geneva	6523.2	1087.2
Nigeria	45	ABV Abuja > FRA Frankfurt > GVA Geneva	32562	723.6
North Korea, Democratic People's Republic of Korea	8	ICN Seoul > WAW Warsaw > GVA Geneva	7387.2	923.4
Norway	25	OSL Oslo > MUC Munich > GVA Geneva	10535	421.4
Oman	10	MCT Muscat > FRA Frankfurt > GVA Geneva	7298	729.8
Pakistan	14	ISB Islamabad > AUH Abu Dhabi > GVA Geneva	11113.2	793.8
Palau	1	ROR Koror > GUM Guam > NRT Tokyo > AUH Abu Dhabi > GVA Geneva	1846.6	1846.6
Palestine	8	TLV Tel Aviv > GVA Geneva	3259.2	407.4
Panama	6	PTY Panama City > CDG Paris > GVA Geneva	5850	975
Paraguay	9	ASU Asunción > GRU Sao Paulo > CDG Paris > GVA Geneva	10701	1189
Peru	13	LIM Lima > AMS Amsterdam > GVA Geneva	15958.8	1227.6
Philippines	14	MNL Manila > IST Istanbul > GVA Geneva	13252.4	946.6
Poland	7	WAW Warsaw > GVA Geneva	2325.4	332.2

Khorsand P, *et al. BMJ Global Health* 2022; 7:e009231. doi: 10.1136/bmjgh-2022-009231

Portugal	13	LIS Lisbon > GVA Geneva	3853.2	296.4
Qatar	26	DOH Hamad > IST Istanbul > GVA Geneva	17882.8	687.8
Republic of Congo	10	FIH Kinshasa > IST Istanbul > GVA Geneva	9660	966
Republic of Korea	53	ICN Seoul > WAW Warsaw > GVA Geneva	48940.2	923.4
Republic of North Macedonia	6	SKP Skopje > VIE Vienna > GVA Geneva	2498.4	416.4
Romania	10	OTP Bucharest > VIE Vienna > GVA Geneva	4162	416.2
Russian Federation	42	SVO Moscow > GVA Geneva	16556.4	394.2
Rwanda	6	KGL Kigali > EBB Entebbe > BRU Brussels > GVA Geneva	3763.2	627.2
Saint Kitts and Nevis	2	SKB Basseterre > MIA Miami > MAD Adolfo > GVA Geneva	2590	1295
Saint Lucia	3	SLU St Lucia Vigie > FDF Fort De France > CDG Paris > GVA Geneva	2975.4	991.8
Saint Vincent and the Grenadines	6	SVD Argyle > BGI Grantley Adams > MIA Miami > LHR London > GVA Geneva	8739.6	1456.6
Samoa	4	APW Apia > AKL Auckland > SIN Singapore > FRA Frankfurt > GVA Geneva	8853.2	2213.3
San Marino	4	BLQ Bologna > LHR London > GVA Geneva	1672.8	418.2
Sao Tome and Principe	3	TMS Sao Tome > LIS Lisbon > GVA Geneva	2631.6	877.2
Saudi Arabia	21	RUH Riyadh > CAI Cairo > GVA Geneva	13931.4	663.4
Senegal	14	DSS Dakar > LIS Lisbon > GVA Geneva	9671.2	690.8
Serbia	5	BEG Belgrade > MUC Munich > GVA Geneva	1849	369.8
Seychelles	5	SEZ Mahe Island > AUH Abu Dhabi > GVA Geneva	4921	984.2
Sierra Leone	12	FNA Freetown > ROB Monrovia Roberts > BRU Brussels > GVA Geneva	7668	639
Singapore	15	SIN Singapore > HEL Helsinki > GVA Geneva	15891	1059.4
Slovakia	12	BTS Bratislava > LGW London > GVA Geneva	3637.2	303.1
Slovenia	5	LJU Ljubljana > FRA Frankfurt > GVA Geneva	1886	377.2
Solomon Islands	5	HIR Honiara > BNE Brisbane > DXB Dubai > GVA Geneva	11861.5	2372.3
Somalia	7	MGQ Mogadishu > ADD Addis Ababa > GVA Geneva	5278	754
South Africa	12	CPT Cape Town > ADD Addis Ababa > GVA Geneva	15520.8	1293.4
South Sudan	6	JUB Juba > ADD Addis Ababa > GVA Geneva	4539.6	756.6
Spain	31	MAD Madrid > GVA Geneva	6925.4	223.4
Sri Lanka	33	CMB Colombo > JED Jeddah > GVA Geneva	37659.6	1141.2

Sudan	18	KRT Khartoum > IST Istanbul > GVA Geneva	12754.8	708.6
Suriname	2	PBM Paramaribo > AMS Amsterdam > GVA Geneva	1928.8	964.4
Sweden	28	ARN Stockholm > GVA Geneva	9044	323
Switzerland	17	N/A	0	
Syrian Arab Republic	6	BEY Beirut > FRA Frankfurt > GVA Geneva	3373.2	562.2
Tajikistan	4	DYU Dushanbe > IST Istanbul > ZRH Zurich > GVA Geneva	2851.6	712.9
Tanzania, United Republic of	31	DOD Dodoma > DAR Dar Es Salaam > IST Istanbul > GVA Geneva	33418	1078
Thailand	60	BKK Bangkok > HEL Helsinki > GVA Geneva	58452	974.2
Timor Leste	8	CBR Canberra > MEL Melbourne > AUH Abu Dhabi > GVA Geneva	13859.2	1732.4
Togo	7	LFW Lomé > ADD Addis Ababa > GVA Geneva	7722.4	1103.2
Tonga	4	TBU Tongatapu > NAN Nan > SFO San Francisco > CDG Paris > GVA Geneva	9008	2252
Trinidad and Tobago	4	POS Port of Spain > YYZ Toronto > LHR London > GVA Geneva	5432.8	1358.2
Tunisia	12	TUN Tunis > GVA Geneva	2772	231
Turkey	25	ESB Ankara > IST Istanbul > GVA Geneva	10615	424.6
Turkmenistan	9	ASB Ashgabat > IST Istanbul > GVA Geneva	5279.4	586.6
Tuvalu	3	FUN Funafuti > SUV Suva > NAN Nan > SFO San Francisco > DUB Dublin > GVA Geneva	6603	2201
Uganda	15	EBB Entebbe > ADD Addis Ababa > GVA Geneva	11652	776.8
Ukraine	10	KBP Kiev > GVA Geneva	4216	421.6
United Arab Emirates	15	AUH Abu Dhabi > TLV Tel Aviv > GVA Geneva	11415	761
United Kingdom	62	BFS Belfast > LTN London > GVA Geneva	20615	332.5
United States of America	50	IAD Washington > LIS Lisbon > GVA Geneva	49380	987.6
Uruguay	4	MVD Montevideo > GRU Sao Paulo > AMS Amsterdam > GVA Geneva	5703.6	1425.9
Uzbekistan	4	TAS Tashkent > IST Istanbul > GVA Geneva	2524.8	631.2
Vanuatu	8	VLI Port Vila > NAN Nan > SFO San Francisco > CDG Paris > GVA Geneva	18035.2	2254.4
Venezuela	13	CCS Caracas > PTY Panama City > FRA Frankfurt > GVA Geneva	18483.4	1421.8
Vietnam	21	HAN Hanoi > BKK Bangkok > HEL Helsinki > GVA Geneva	24162.6	1150.6
Yemen	6	MCT Muscat > FRA Frankfurt > GVA Geneva	4378.8	729.8
Zambia	28	LUN Lusaka > JNB Johannesburg > ZRH Zurich > GVA Geneva	40264	1438
Zimbabwe	27	HRE Harare > JNB Johannesburg > ZRH Zurich > GVA Geneva	38121.3	1411.9

Khorsand P, *et al. BMJ Global Health* 2022; 7:e009231. doi: 10.1136/bmjgh-2022-009231
